# Supplementary material for: URJC-1: Stable and Efficient Catalyst for O-Arylation Cross-Coupling
Source: Nanomaterials (Basel). 2024 Jun 27;14(13):1103. doi: 10.3390/nano14131103 (PMC11243573; doi:10.3390/nano14131103)
Supplement: Supplementary file 1 [file nanomaterials-14-01103-s001.zip › nanomaterials-2946647-supplementary.pdf]

URJC-1: Stable and Efficient Catalyst for O-Arylation Cross-Coupling

Elena García-Rojas, Pedro Leo *, Jesús Tapiador, Carmen Martos and Gisela Orcajo

Chemical and Environmental Engineering Group, ESCET, Rey Juan Carlos University, C/Tulipán s/n 28933 Móstoles, Spain

*Correspondence: pedro.leo@urjc.es

SUPLEMENTARY INFORMATION

S1. Characterization of Cu-URJC-1.

S2. Characterization of Cu-MOF-74.

S3. Characterization of JUC-62.

S4. Characterization of HNUST-1.

S5. Characterization of HKUST-1.

S6. Identification solid product obtained after reaction without MOF.

S7. XRD before and after reaction

S8. Analysis of reaction.

S1. Characterization of Cu-URJC-1.

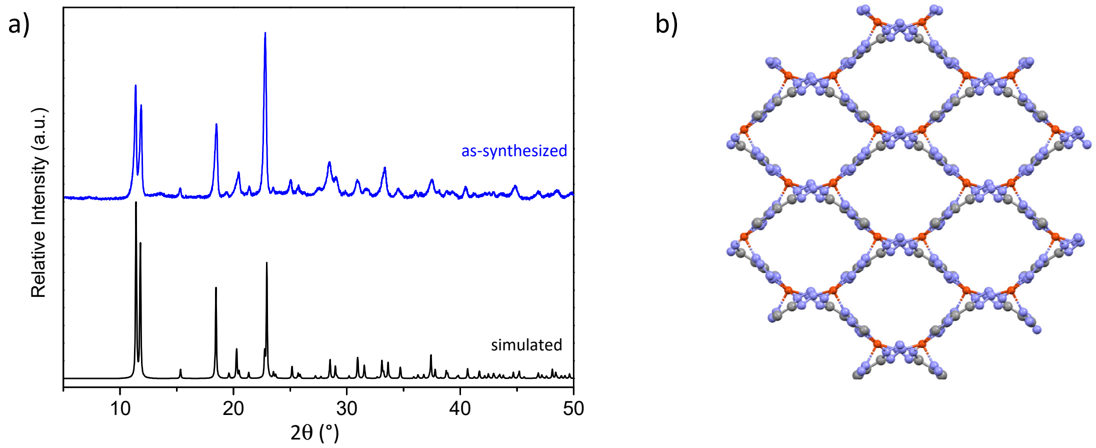

Figure S1.1. a) PXRD of Cu-URJC-1 material, b) channels along the crystallographic axes a.

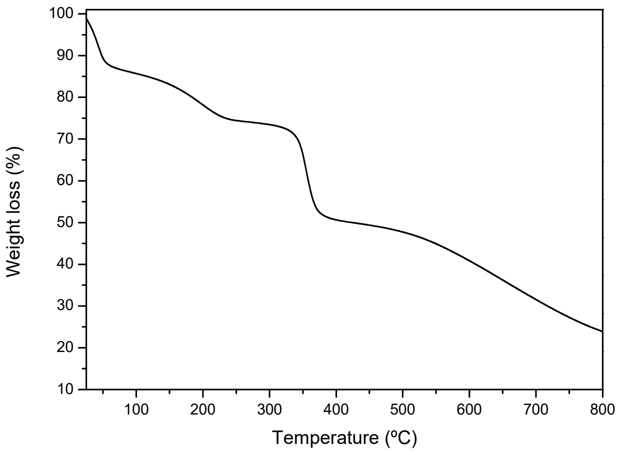

Figure S1.2. TGA of Cu-URJC-1.

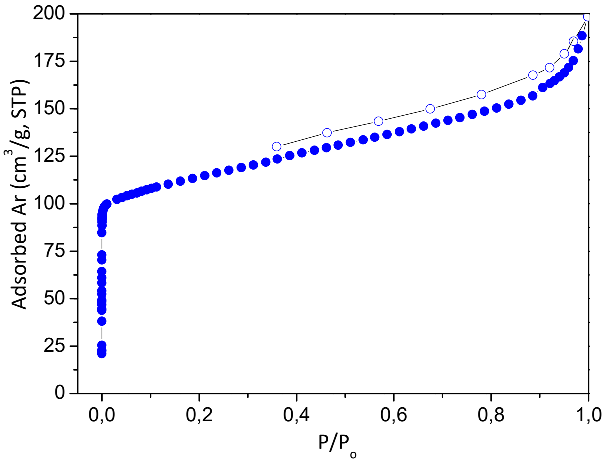

Figure S1.3. Ar adsorption/desorption isotherms at 87K of Cu-URJC-1.

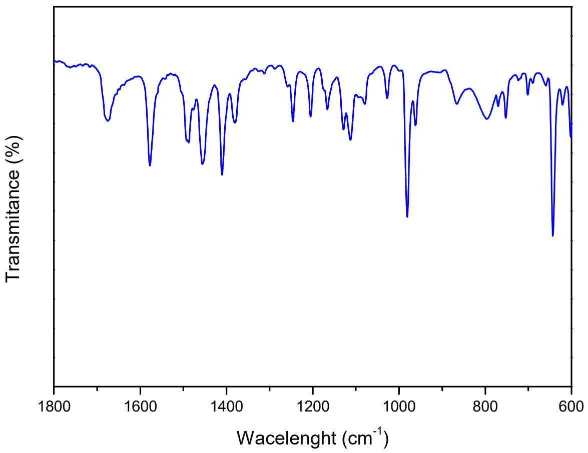

Figure S1.4. IR of Cu-URJC-1.

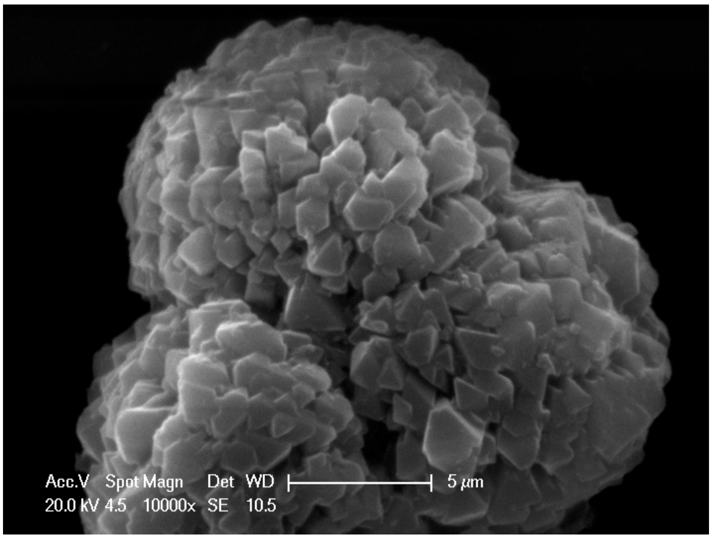

Figure S1.5. SEM image of Cu-URJC-1.

S2. Characterization of Cu-MOF-74.

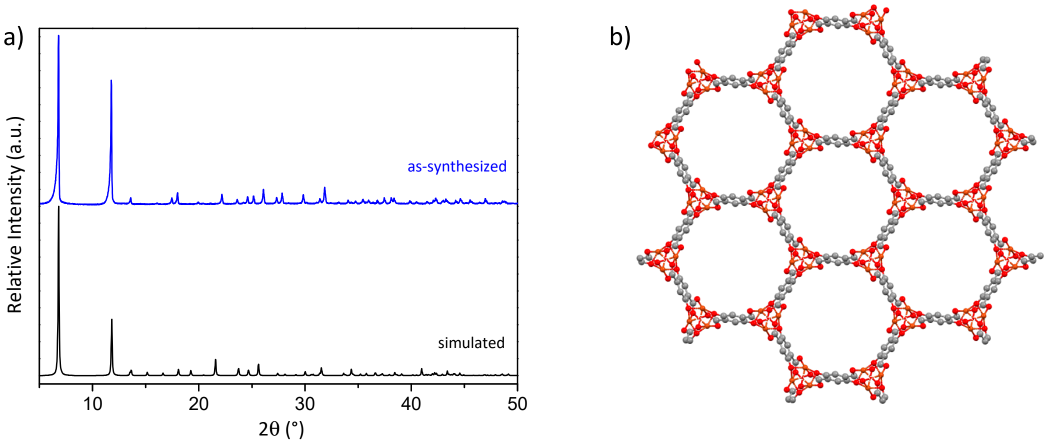

Figure S2.1. a) PXRD of Cu-MOF-74 material, b) channels along the crystallographic axes c.

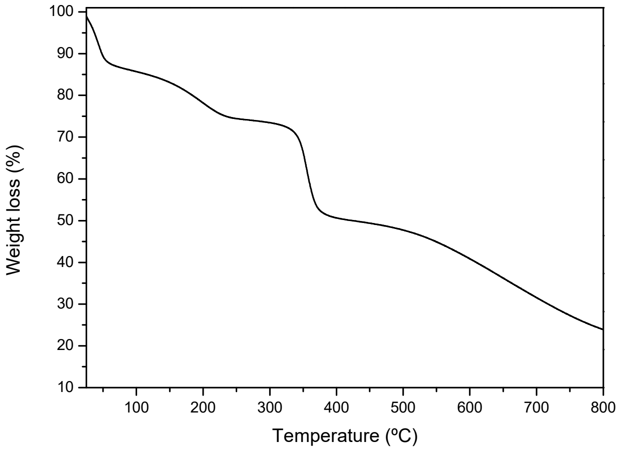

Figure S2.2. TGA of Cu-MOF-74.

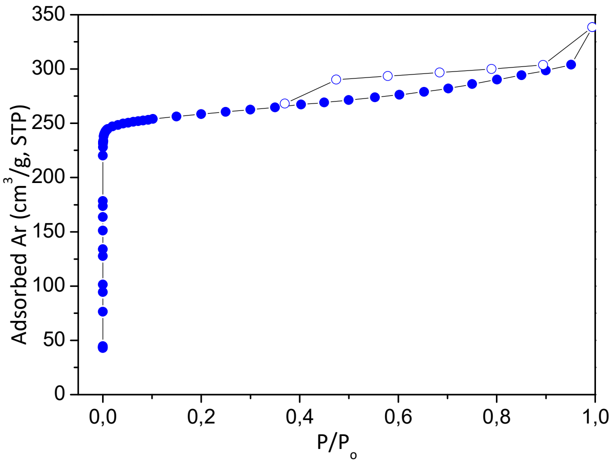

Figure S2.3. Ar adsorption/desorption isotherms at 87K of Cu-MOF-74.

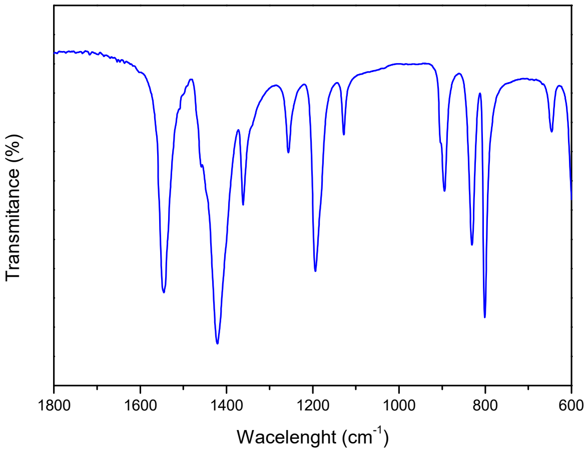

Figure S2.4. IR of Cu-MOF-74.

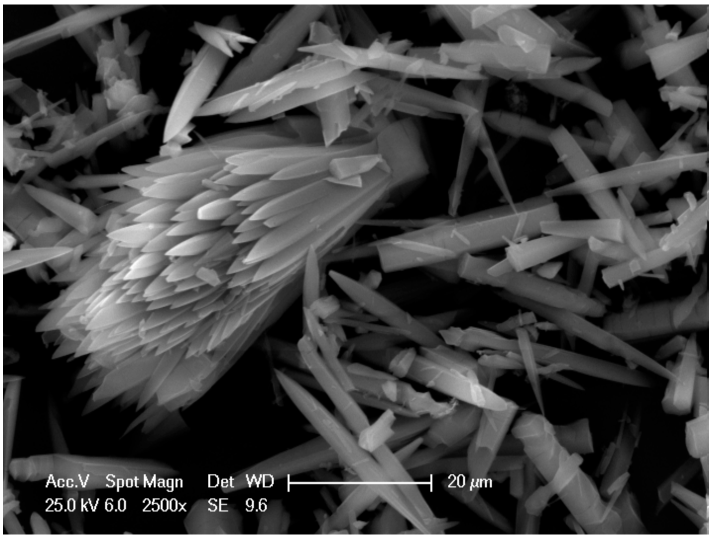

Figure S2.5. SEM image of Cu-MOF-74.

S3. Characterization of JUC-62.

a)

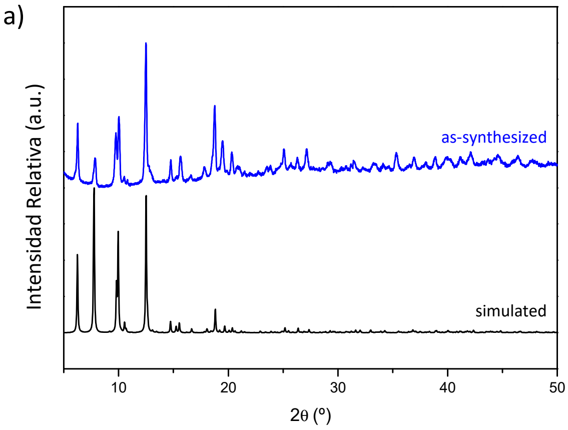

b)

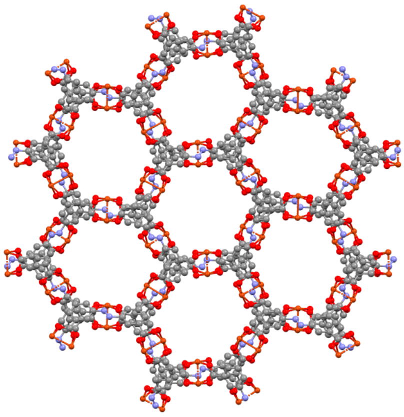

Figure S3.1. a) PXRD of JUC-62 material, b) channels along the crystallographic axes c.

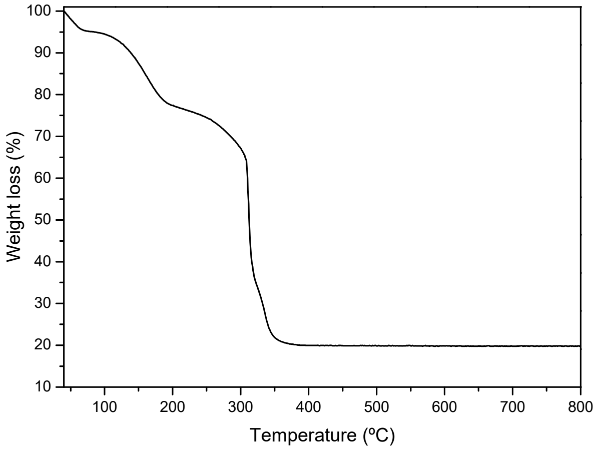

Figure S3.2. TGA of JUC-62.

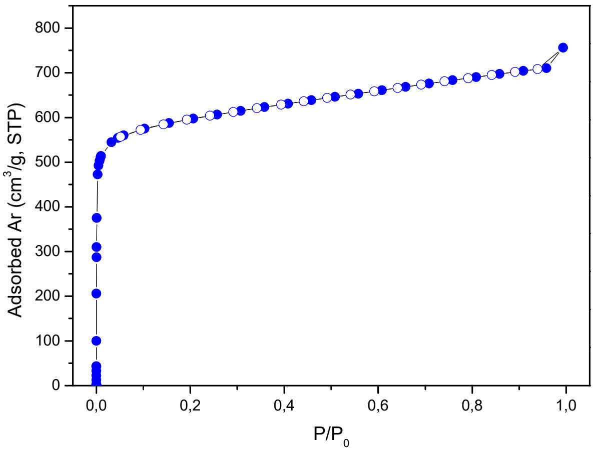

Figure S3.3. Ar adsorption/desorption isotherms at 87K of JUC-62.

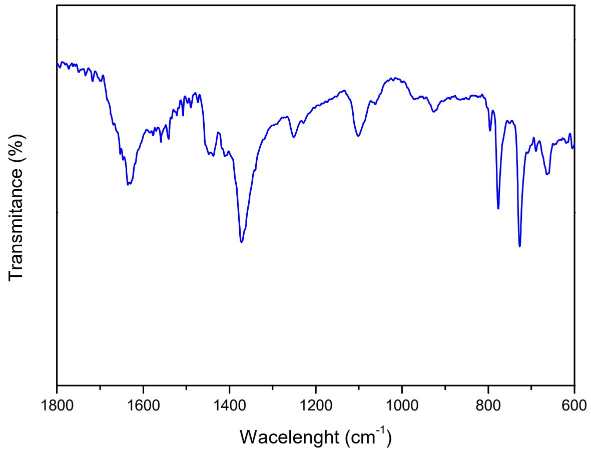

Figure S3.4. IR of JUC-62.

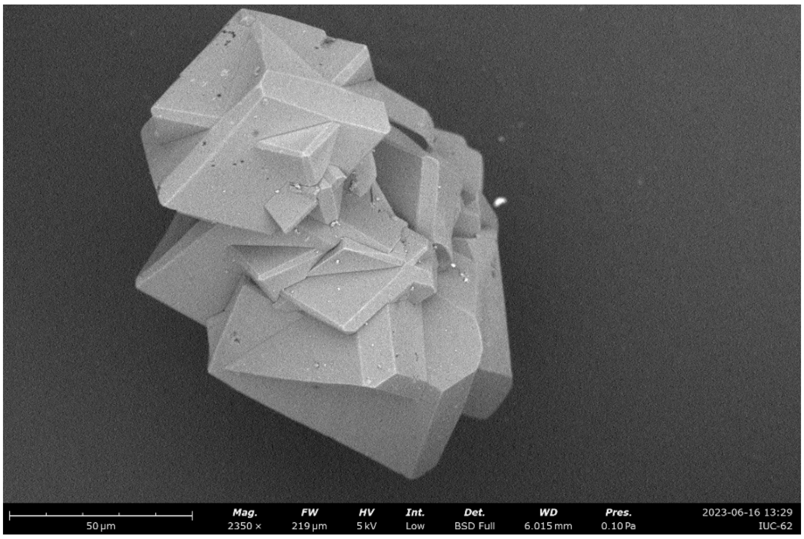

Figure S3.5. SEM image of JUC-62.

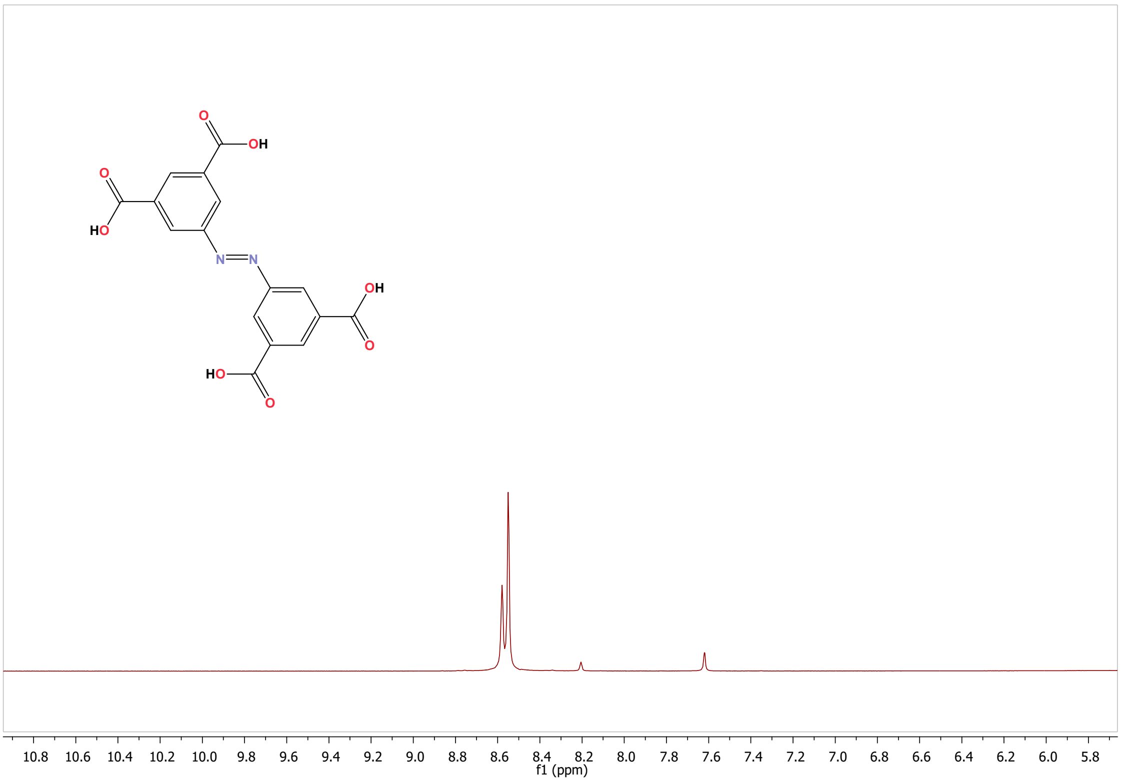

Figure S3.6. ^{1}H NMR of organic linker of JUC-62.

S4. Characterization of HNUST-1.

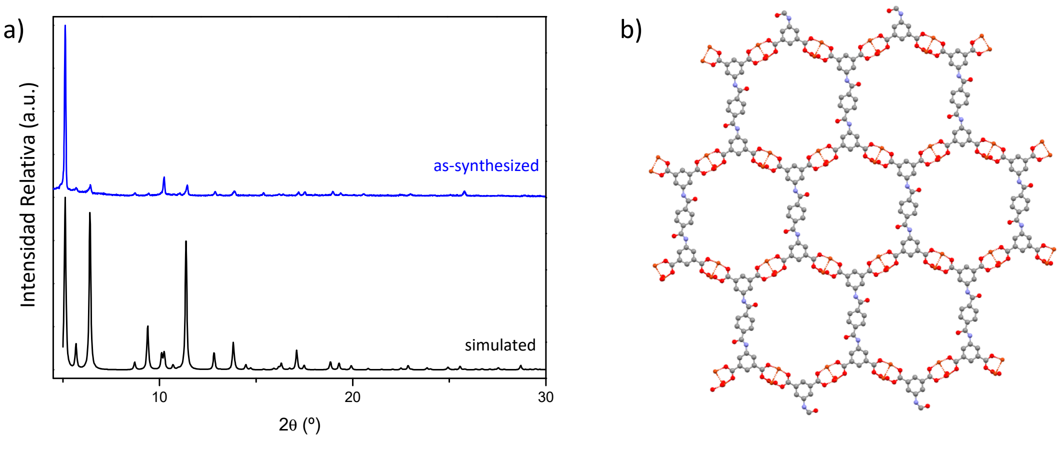

Figure S4.1. a) PXRD of HNUST-1material, b) channels along the crystallographic axes c.

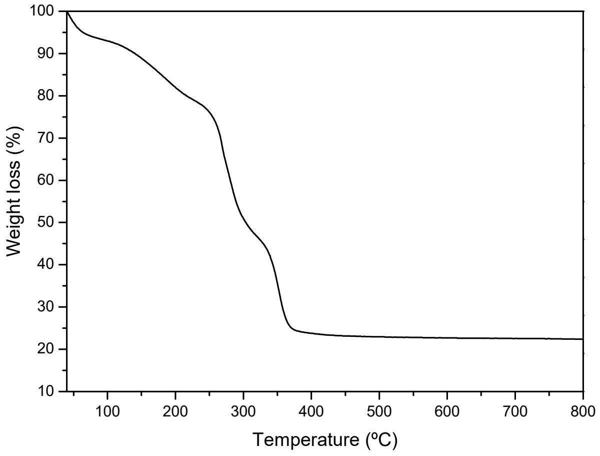

Figure S4.2. TGA of HNUST-1.

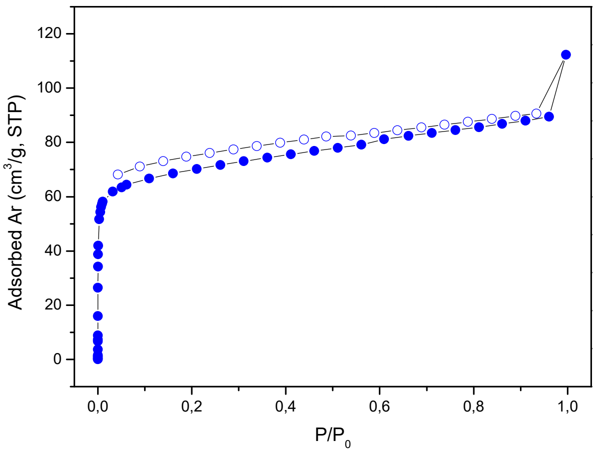

Figure S4.3. Ar adsorption/desorption isotherms at 87K of HNUST-1.

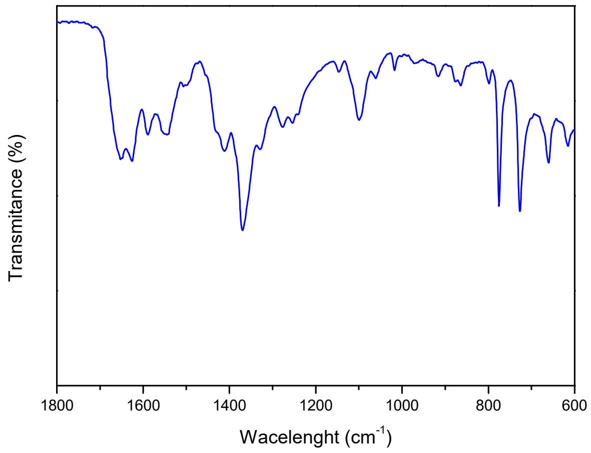

Figure S4.4. IR of HNUST-1.

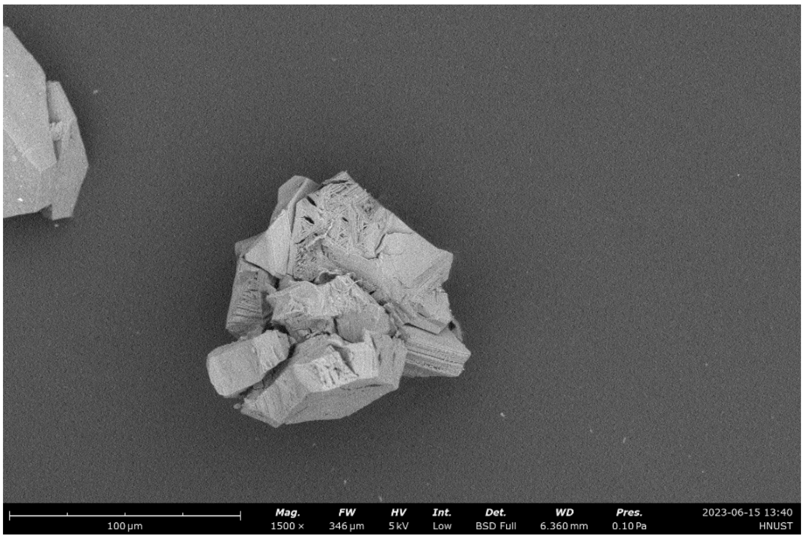

Figure S4.5. SEM image of HNUST-1.

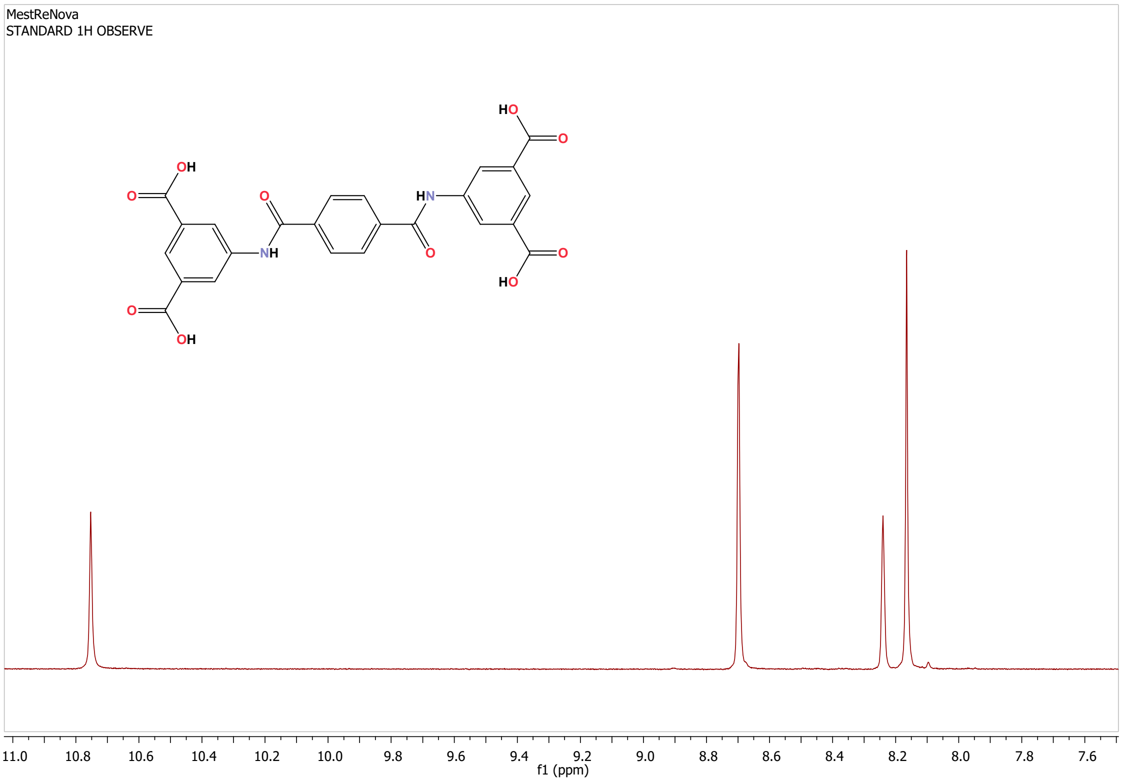

Figure S4.6. ^{1}H NMR of organic linker of HNUST-1.

S5. Characterization of HKUST-1.

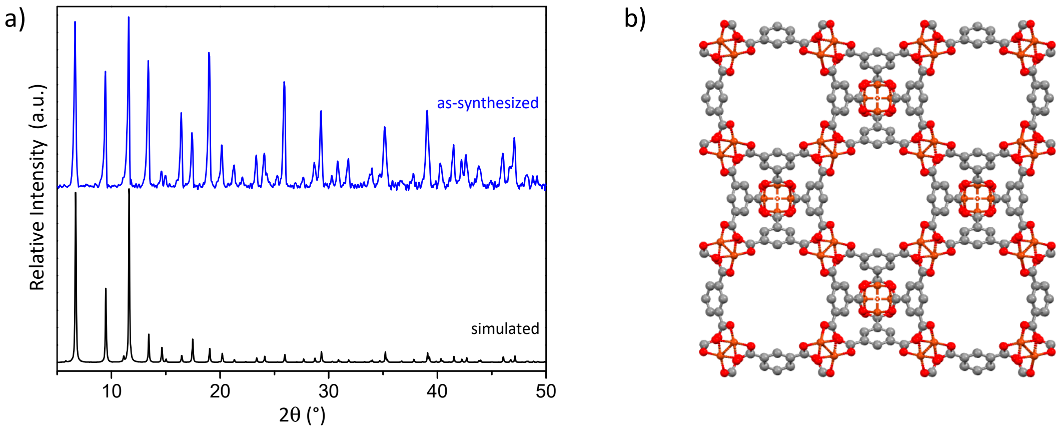

Figure S5.1. a) PXRD of HKUST-1 material, b) channels along the crystallographic axes c.

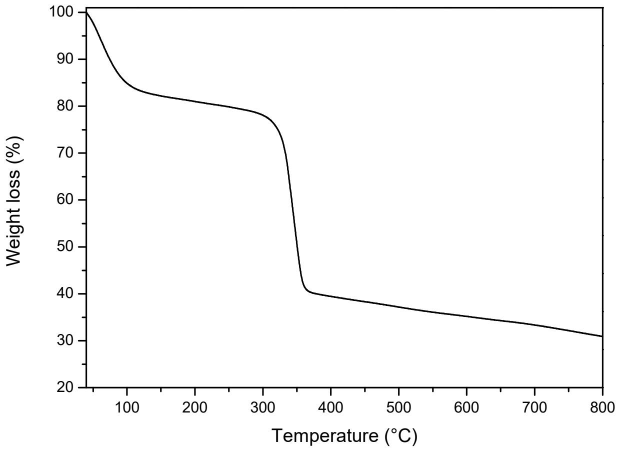

Figure S5.2. TGA of HKUST-1.

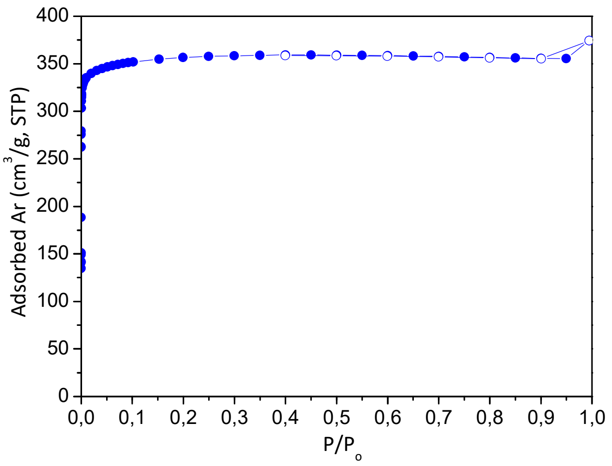

Figure S5.3. Ar adsorption/desorption isotherms at 87K of HKUST-1.

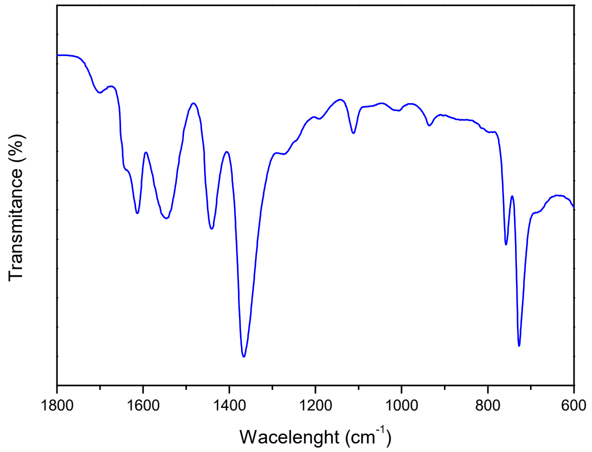

Figure S5.4. IR of HKUST-1.

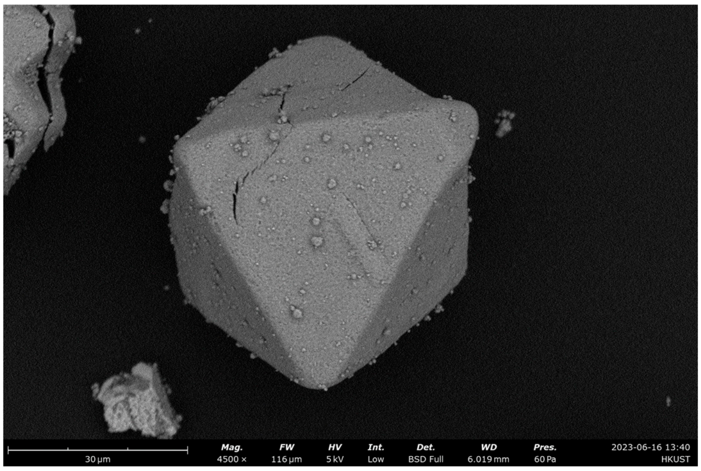

Figure S5.5. SEM image of HKUST-1.

S6. Identification solid product obtained after reaction without MOF.

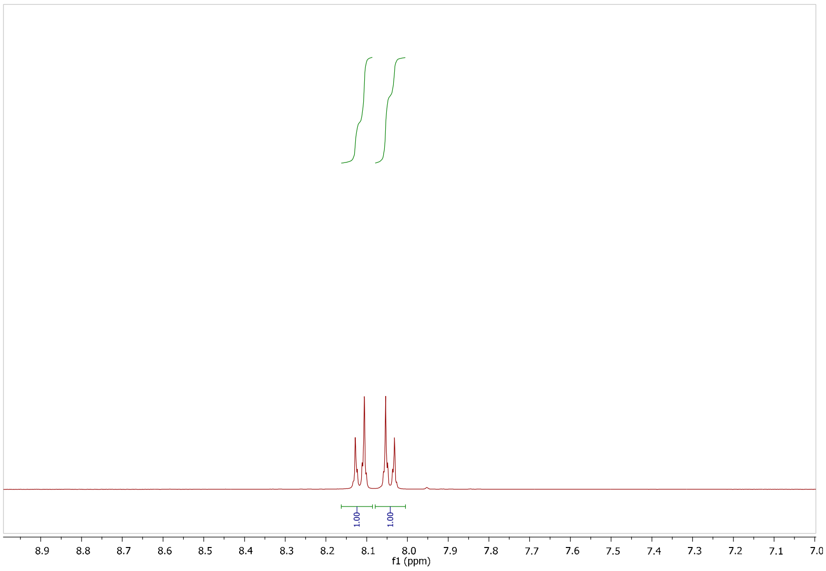

Figure S6.1. ^{1}H NMR of solid product obtained after reaction without catalyst.

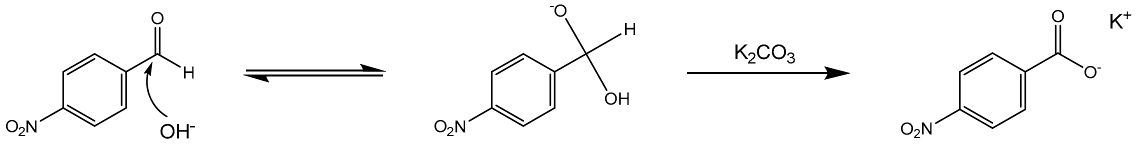

Figure S6.2. Scheme of Cannizzaro reaction in presence of K_2CO_3.

S7. XRD before and after reaction

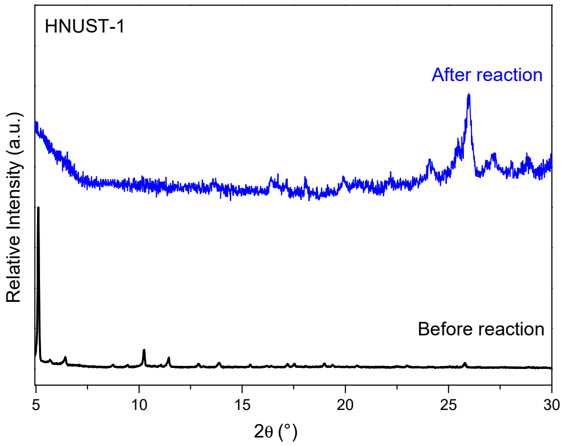

Figure S7.1 XRD of HNUST-1.

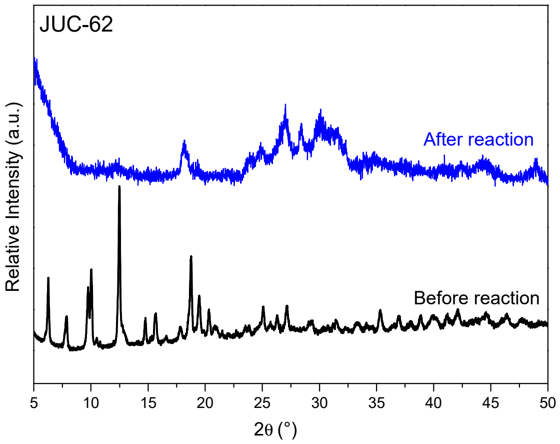

Figure S7.2 XRD of JUC-62.

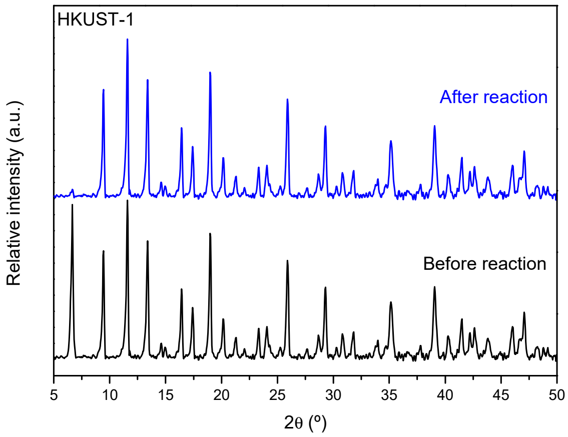

Figure S7.3 XRD of HKUST-1.

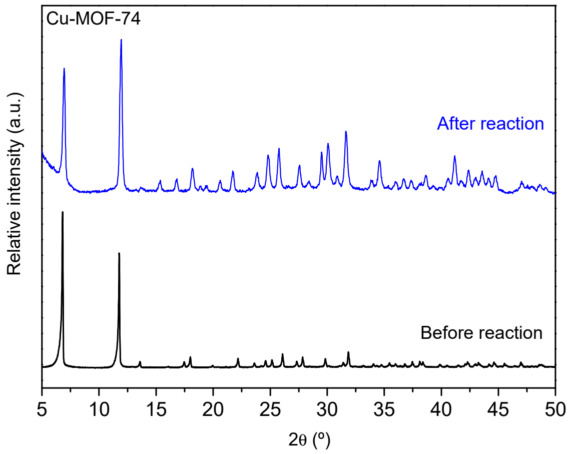

Figure S7.4 XRD of Cu-MOF-74.

S8. Analysis of reaction.

All reagents and products used in the reaction were purchased with high purity. Each spice was injected individually into the GC in order to identify the times at which they appeared. A calibration line was prepared to relate the peak area to the mass of each species using sulfolane as an internal standard. This methodology permitted the calculation of yield values and the closure of the balance of species in the reaction.

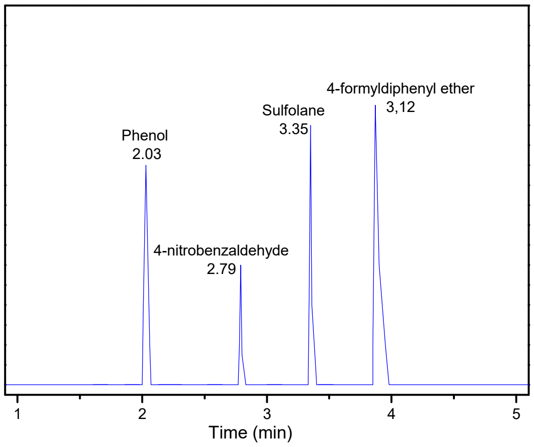

S8.1 Reaction type chromatogram.
